# Supplementary material for: Lipid Raft Integrity and Cellular Cholesterol Homeostasis Are Critical for SARS-CoV-2 Entry into Cells
Source: Nutrients. 2022 Aug 19;14(16):3417. doi: 10.3390/nu14163417 (PMC9415163; doi:10.3390/nu14163417)
Supplement: Supplementary file 1 [file nutrients-14-03417-s001.zip › nutrients-1826914-supplementary.pdf]

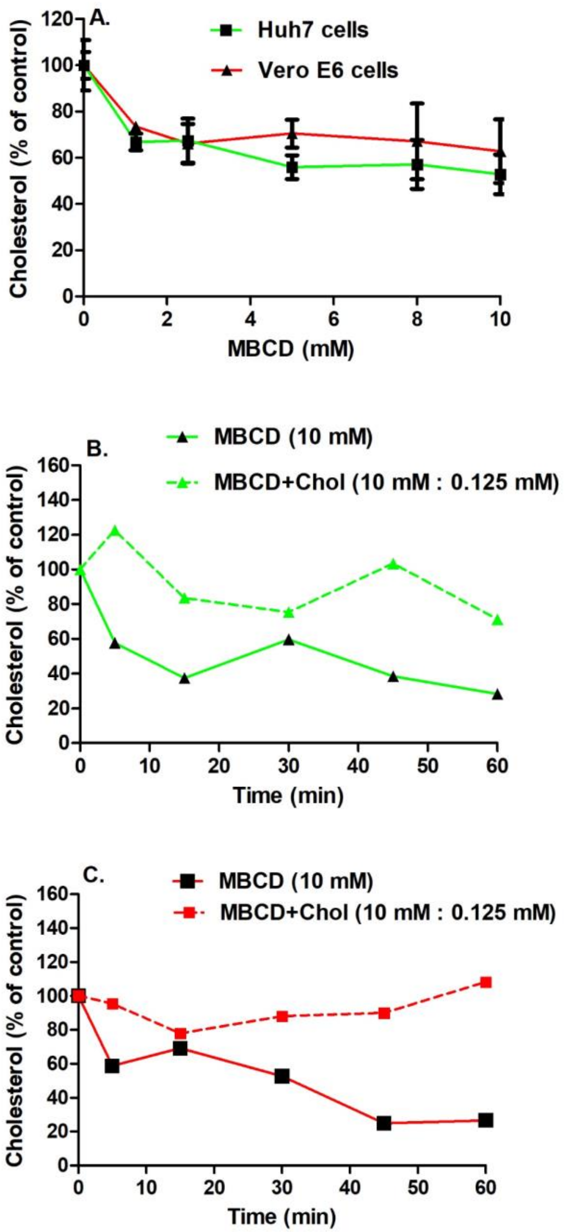


**Figure S1. Effects of cholesterol-depleting agent MBCD on the content of intracellular cholesterol.** Huh7 and Vero E6 cells were grown in DMEM containing 10% FBS until confluency, and then treated with increasing concentrations of MBCD for 1h to deplete cholesterol from cells (**Panel A**, N=3). In subsequent experiments, Huh7 (**Panel B**, N=2) and Vero E6 cells (**Panel C**, N=2) were treated with either BMCD alone (10 mM) or MBCD-cholesterol complexes (10 mM- 0.125 mM) for 5min-60 min. After the treatment, total cholesterol was extracted from the cells and measured by colorimetric cholesterol assay.

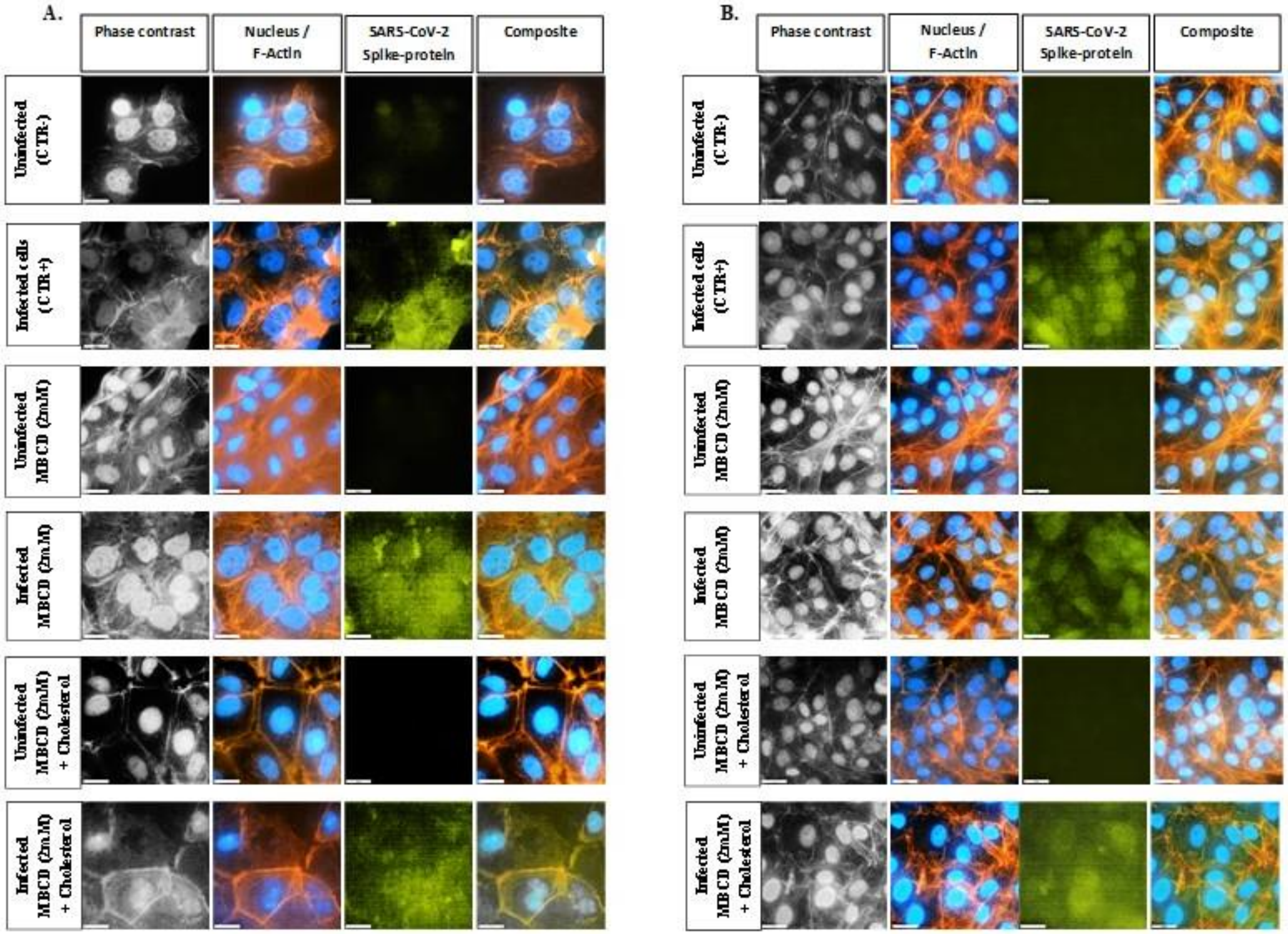


**Figure S2. MβCD treatment did not disturb the cell morphology in wild-type SARS-CoV-2 infected cells.** Huh7 and Vero E6 cells were treated with MβCD (2mM) or MBCD + cholesterol for 1h prior infection with wild-type SARS-Co-V2pp (MOI=0.1). At 48h p.i., Huh7 (**Panel A**) and Vero E6 cells (**Panel B**) were fixed and analyzed by immunofluorescence staining using DAPI for cell nuclei (blue), anti-actin antibody (orange), SARS-Co-V2 anti-spike proteins (green) and merged double stained (composite). MβCD-treated cells were compared to controls: untreated-uninfected (CTR-) and untreated-infected (CTR+) cells. Images were taken and analyzed with resolution of fluorescence microscopy up to 20μm.

**Table S1.** Primers and probes used to detect SARS-CoV-2

| Gene | Primer/Probe | Sequence (5'–3') |
|---|---|---|
| RdRp-gene | RdRP_SARSr-F2<br>RdRP_SARSr-R1<br>RdRP_SARSr-P2<br>RdRP_SARSr-P1 | GTGARATGGTCATGTGTGGCGG<br>CARATGTTAAASACACTATTAGCATA<br>FAM CAGGTGGAACCTCATCAGGAGATGC-BBQ<br>FAM-CCAGGTGWACRTCATCMGGTGATGC-BBQ |
| E-gene | E_Sarbeco_F1<br>E_Sarbeco_R2<br>E_Sarbeco_P1 | ACAGGTACGTTAATAGTTAATAGCGT<br>ATATTGCAGCAGTACGCACACA<br>FAM-ACACTAGCCATCCTTACTGCGCTTCG-BBQ |
| N-gene | N_Sarbeco_F1<br>N_Sarbeco_R1<br>N_Sarbeco_P1 | CACATTGGCACCCGCAATC<br>GAGGAACGAGAAGAGGCTTG<br>FAM-ACTTCCTCAAGGAACAACATTGCCA-BBQ |

R, A/G; W, A/T; FAM, 6-fluorescein amidite; BBQ, blackberry quencher

**Table S2.** List of primers used for qRT-PCR

| Target Gene (Human) | Primer Sequences (5′-3′) |
|---|---|
| CH25H | F: CTTTCCGTGGAGGACCACTC<br>R: GTGAGAGTGATGCAGGTCGT |
| SREBF1 | F: CAGCAGCTACTGACAGTCACA<br>R: CTTGATGAAGTGGGGCTGC |
| SCAP | F: TGCACTGAACCTGGACTTGG<br>R: CCCAAAGTGCCTGACAGATG |
| LDLR | F: CGTGCTCCTCGTCTTCCTTT<br>R: TCTGTCTCGAGGGGTAGCTG |
| ACAT1 | F: GTTCTGGTCCAAAAACCGCC<br>R: CGCCGAGACCTTGGAAGTAG |
| HMGCR | F: CTCTTATTGGTCGAAGGCTCG<br>R: CCACAAAGAGGCCATGCATTC |
| HMGCS1 | F: GGGCGTTGAGGTCTAGGTATTCT<br>R: TTTCCTCCTTCGGGCACTCTA |
| APOA1 | F: CTGTTTGCCCACTCTATTTGCC<br>R: CCTGTTGCTGCTCACTGGTC |
| APOB | F: ACTGCTAAAGGCATGGCACT<br>R: TGCCGTGATCTCAAATGGCT |
| SREBPF2 | F: TCTGGAGACCATGGAGACCC<br>R: GTCAGGGAACTCTCCCACTTG |
| NPC1L1 | F: TGAGCTGCATGGCTGACTAC<br>R: AGGGCCTCTGCCTCAGAATA |
| PPARA | F: GCGAACGATTCGACTCAAGC<br>R: CCAGGACGATCGTTGTGTGA |
| PPARG | F: CCAGAAGCCTGCATTTCTGC<br>R: CACGGAGCTGATCCCAAAGT |
| PPARD | F: CCGGGACAGTGTTGTACAGT<br>R: AGGTCTCGTTGGTGCATCTG |
| FDFT1 | F: GCATGAGCGACTTTTGCGTG<br>R: GCCTGGATAACAGCTGCGAA |
| NR1H2 | F: TGTCCTCTCCTACCACGAGT<br>R: TTCAGAAAGGACGCCCCAGT |
| NR1H3 | F: AGCCAAGGTACAGGTAACGA<br>R: GGTTCCAGCTTGTTTGCTGC |
| MBTPS1 | F: GGACCAGTGTTGCTTCTCCA<br>R: CTGCTTCATACTGGCGGGAT |
| MBTPS2 | F: TGTCGTCTACCTGACCGACT<br>R: TCCGTTGTTTTCCAGCCAGT |
| ACAT2 | F: GACTTCGTCTCCTTCGTGCC<br>R: CCCCAAATCCGAAGGACTGG |
| ACE2 | F: GGGATCAGAGATCGGAAGAAGAAA<br>R: AGGAGGTCTGAACATCATCAGTG |
| ABCA1 | F: GGGTGGTGTTCTTCCTCATTACTG<br>R: CCGCCTCACATCTTCATCTTCATC |
| ACTIN | F: ATCGTGCGTGACATTAAGGAGAAG<br>R: AGGAAGGAAGGCTGGAAGAGTG |

**F**, forward; **R**, reverse
